# Hspa13 Promotes Plasma Cell Production and Antibody Secretion

**DOI:** 10.3389/fimmu.2020.00913

**Published:** 2020-05-29

**Authors:** Youdi He, Ruonan Xu, Bing Zhai, Ying Fang, Chunmei Hou, Chen Xing, He Xiao, Guojiang Chen, Xiaoqian Wang, Ning Ma, Gencheng Han, Renxi Wang

**Affiliations:** ^1^Beijing Institute of Brain Disorders, Laboratory of Brain Disorders, Ministry of Science and Technology, Collaborative Innovation Center for Brain Disorders, Capital Medical University, Beijing, China; ^2^Department of Neurology, Beijing Chaoyang Hospital, Capital Medical University, Beijing, China; ^3^Department of Geriatric Hematology, Chinese PLA General Hospital, Beijing, China; ^4^Department of Rheumatology, First Hospital of Jilin University, Changchun, China; ^5^Institute of Military Cognition and Brain Sciences, Beijing, China; ^6^State Key Laboratory of Toxicology and Medical Countermeasures, Institute of Pharmacology and Toxicology, Beijing, China; ^7^Staidson (Beijing) Biopharmaceuticals Co., Ltd, Beijing, China

**Keywords:** Hspa13, SLE, B cells, plasma cells, antibody

## Abstract

The generation of large numbers of plasma cells (PCs) is a main factor in systemic lupus erythematosus (SLE). We hypothesize that Hspa13, a member of the heat shock protein family, plays a critical role in the control of PC differentiation. To test the hypothesis, we used lipopolysaccharide (LPS)-activated B cells and a newly established mouse line with a CD19^cre^-mediated, B cell–specific deletion of Hspa13: Hspa13 cKO mice. We found that Hspa13 mRNA was increased in PCs from atacicept-treated lupus-prone mice and in LPS-stimulated plasmablasts (PBs) and PCs. A critical finding was that PBs and PCs [but not naïve B cells and germinal center (GC) B cells] expressed high levels of Hspa13. In contrast, the Hspa13 cKO mice had a reduction in BPs, PCs, and antibodies induced *in vitro* by LPS and *in vivo* by sheep red blood cells (SRCs)- or 4-hydroxy-3-nitrophenylacetyl (NP)-immunization. Accordingly, the Hspa13 cKO mice had reduced class-switched and somatically hypermutated antibodies with defective affinity maturation. Our work also showed that Hspa13 interacts with proteins (e.g., Bcap31) in the endoplasmic reticulum (ER) to positively regulate protein transport from the ER to the cytosol. Importantly, Hspa13 mRNA was increased in B220^+^ cells from patients with multiple myeloma (MM) or SLE, whereas Hspa13 cKO led to reduced autoantibodies and proteinuria in both pristane-induced lupus and lupus-prone MRL/lpr mouse models. Collectively, our data suggest that Hspa13 is critical for PC development and may be a new target for eliminating pathologic PCs.

## Introduction

Plasma cells (PCs) play a critical role in the immune response by producing antibody (1, 2). B cells arise in the bone marrow and mature and differentiate into germinal center (GC) B cells, plasmablasts (PBs), and terminally differentiated PCs in the peripheral secondary lymphoid tissues such as the spleen and lymph nodes (LNs) (3–5). The process of the differentiation of B cells into PCs is regulated by some important transcriptional factors, including the PC-inhibitory factors Pax5 and Bcl6 and the PC-promoting factors Prdm1 (Blimp1) and Xbp1 (3–7).

The abnormal production of PCs is involved in the pathology of both multiple myeloma (MM) and systemic lupus erythematosus (SLE). MM is a malignancy of PCs; patients typically present with an infiltration of the bone marrow with clonal PCs and monoclonal protein in the serum and/or urine (8–10). Most cases of MM, especially in relapsed patients, are incurable (11). Autoreactive PCs and pathogenic autoantibodies are critical factors involved in SLE pathology (12, 13). Belimumab, a human anti-BAFF (B-cell activation factor) antibody that selectively depletes mature and activated B cells and PBs and results in an increase in the number of PCs, has been used to treat patients with SLE (14, 15). The drug atacicept (TACI-IgG) is a recombinant fusion protein containing the extracellular ligand-binding protion of human TACI (transmembrane activator and calcium modulator and cyclophilin-ligand interactor, one of the BAFF receptors) linked to the Fc fragment of human IgG; its effects are similar to those of belimumab (16, 17). These results suggest there is no effective curative treatment for MM or SLE that targets PCs.

The binding immunoglobulin protein (BiP), also known as GRP-78, heat shock 70-kDa protein 5 (HSPA5), or (Byun1), is the first chaperone discovered that non-covalently binds to free IgH but not to IgH associated with IgL (18). Heat shock proteins (HSPs) (e.g., Hsp90) ensure correct protein folding (e.g., antibody) in PCs and cell survival. Stressful conditions often stimulate cells to produce HSPs (19, 20). HSPs interact with cellular proteins to ensure proper protein folding and transport from the endoplasmic reticulum (ER) into the cytoplasm or secretory pathway (21–23). HSPs also contribute to protein (e.g., HSP) misfolding that mediates amyloid β oligomer accumulation (24, 25). HSP (e.g., Hsp90) inhibitors induce the unfolded protein response (UPR) to reduce abnormal immunoglobulin production and cell death (26). These results suggest that targeting HSPs may represent a novel therapeutic strategy for controlling abnormal PCs.

We hypothesize that Hspa13, a member of the heat shock protein family, plays a critical role in the control of PC differentiation. To test the hypothesis, we used lipopolysaccharide (LPS)-activated B cells and a newly established mouse line with a CD19^cre^-mediated, B cell-specific deletion of Hspa13 (Hspa13 cKO). We found that PBs and PCs (but not naïve B cells and GC B cells) expressed high levels of Hspa13. In contrast, the Hspa13 cKO mice had a reduction in PBs, PCs, and antibodies induced *in vitro* by LPS and *in vivo* by sheep red cells (SRCs) or 4-hydroxy-3-nitrophenylacetyl (NP)-immunization, and there were reduced numbers of autoantibodies and levels of proteinuria in both pristane-induced lupus and lupus-prone MRL/lpr mouse models. Collectively, our data suggest that Hspa13 is critical for PC development and may be a new target for eliminating pathologic PCs.

## Methods and Materials

### Ethics Committee Approval

Care, use, and treatment of mice in this study were in strict agreement with international guidelines for the care and use of laboratory animals. This study was approved by the Animal Ethics Committee of the Beijing Institute of Basic Medical Sciences.

### Mice and Immunization

Seven-to-nine-week-old C57BL/6, Balb/c (Huafukang Corp., Beijing, China), female lupus-prone MRL/MpJ/lpr/lpr (MRL/lpr) mice (Nanjing Biomedical Research Institute of Nanjing University, Nanjing, China) have been previously described (27). The floxed Hspa13 (Hspa13^fl/fl^) mice in a B6 background were generated by Shanghai Biomodel Organism Science & Technology Development Co., Ltd. (Shanghai, China). To delete Hspa13 in B cells, Hspa13^fl/fl^ mice were crossed with heterologous CD19^cre^ mice to generate CD19^cre^Hspa13^fl/fl^ (Hspa13 cKO) mice. Wild type (WT), Hspa13^fl/fl^, and heterologous CD19^cre^ mice were used as the control for Hspa13 cKO mice.

Three lupus-prone MRL/lpr mice per group were injected intraperitoneally (i.p.) with 5 mg/kg atacicept (TACI-IgG) and control (IgG) at 1, 2, 3, and 4 weeks (two times per week) after mice reached 6 months of age based on a previous protocol (28).

Hspa13 cKO and control mice were injected i.p. with 1 × 10^9^ sheep red cells (SRCs, Hongquan Bio, Beijing, China), or 100 μg of 4-Hydroxy-3-nitrophenylacetyl (NP)-Ficoll or NP-Keyhole Lymphocyte Hemocyanin (KLH) (Biosearch Technologies) in alum on day 0 and then boosted i.p. with the same reagent on day 7.

To explore the role of Hspa13 in lupus, the floxed Hspa13 (Hspa13^fl/fl^) mice in lupus-prone MRL/lpr mice background were generated and crossed with CD19^cre^ mice to generate CD19^cre^Hspa13^fl/fl^ (Hspa13 cKO) mice.

### Peripheral Blood From Normal Human Subjects, Patients With Multiple Myeloma (MM), and Patients With Systemic Lupus Erythematosus (SLE)

Blood samples were obtained after the approval from the Beijing Institute of Basic Medical Sciences, consent from 9 normal human subjects, 3 patients with MM, and 6 patients with SLE from Clinical Trial Center (Beijing 301 Hospital). CD19^+^ B cells were isolated using human CD19 MicroBeads (Cat No. 130-090-880, Miltenyi Biotec).

### B-Cell Separation and Culture

B-cell purification and *in vitro* differentiation were previously described (29, 30). Briefly, splenic B220^+^ B cells were separated by B220 microbeads (Cat No. 130-049-501, Miltenyi Biotec). B cells were stimulated with 10 μg/ml LPS (Sigma L2630 from Escherichia coli 0111:B4; Sigma, St Louis, MO) in RPMI 1640 medium containing 10% FBS, 2 mM glutamine, penicillin (100 IU/ml), streptomycin (100 μg/ml), and 50 mM 2-mercaptoethanol.

### Affymetrix Microarrays

Affymetrix microarrays were done based on a previous method (31). Total RNA was extracted from B cells with Trizol and purified over Qiagen RNeasy columns (Qiagen). Synthesis and labeling of RNA and hybridization of arrays were conducted. Stained arrays (430 2.0) were scanned on an Agilent Gene Array Scanner (Affymetrix).

### RNA-Sequencing

The transcripts in cells were determined by RNA-sequencing using previous methods (32–34). Briefly, RNeasy Mini Kit (Qiagen, Venlo, Netherlands) was used to isolate and purify total RNA from cells. NanoDrop®ND-1000 spectrophotometer and Agilent 2100 Bioanalyzer and RNA 6000 NanoChips (Agilent, Palo Alto, CA, USA) were used to determine RNA concentration and quality, respectively. TruSeq Stranded Total RNA Library Prep Kit with Ribo-Zero Gold (Illumina) was used to prepare Libraries. Transcripts were analyzed by RNA-sequencing (Genewiz Corp., Suzhou, China).

### Quantitative PCR (qPCR) Analysis

Total RNA was extracted from cells with Trizol (Invitrogen Life Technologies). The final RNA pellets were dissolved in 0.1 mM EDTA (2 μl/mg original wet weight). Reverse transcription reactions were carried out on 22 μl of sample using superscript II RNA H-Reverse Transcriptase (Invitrogen Life Technologies) in a reaction volume of 40 μl. All samples were diluted in 160 μl nuclease-free water. qPCR was employed to quantify mouse gene expression from the cDNA samples. Mouse gene expression was normalized to the levels of the β-actin gene.

### Western Blot Analysis

Twenty-five microgram of cell protein from whole-cell lysates was electrophoretically separated on a 10% SDS–polyacrylamide gel and then transferred to a PVDF membrane. This membrane was blocked for 1 h in the solution with 5% fat-free dry milk in Tris-buffered saline containing 0.1% Tween-20 (TBS-T) at room temperature. The blots were then incubated overnight at 4°C with rabbit antibodies against Hspa13 (Cat no. 12667-2-AP, Proteintech Group Inc.) and β-tubulin (KM9003T, SunGene Biotech) antibodies diluted 1:1,000 in TBS-T containing 5% bovine serum albumin. The membrane was washed for 5 min every time and totally for 4 times with TBS-T, and incubated for 45 min at room temperature with HRP (horseradish peroxidase)-conjugated secondary antibody F(ab′)2 (Zymed Laboratories, San Francisco, CA) (1:20 000 in TBS-T containing 5% bovine serum albumin). Finally, the ECL detection system (Amersham, Arlington Heights, IL) was used to show the protein band.

### Single-Cell RNA-Sequencing and VDJ-Sequencing

For single-cell capture and library preparation, cells were resuspended in PBS containing 0.04% bovine serum albumin (BSA) (Ambion, Foster City, CA, USA) to a final concentration of 200 cells per μL. This cell suspension was used as an input for automated single-cell capture and barcoding using the 10 X Genomics Full Chromium platform. Single-cell RNA-sequencing and VDJ-sequencing were done by Emei Tongde Corp., Beijing, China.

### Cell Staining, Flow Cytometric Analysis, and Cell Sorting

All cell experiments were strictly prepared on ice, unless otherwise stated in other specific procedures. Cells (1 × 10^6^ cells/sample) were washed with fluorescence-activated cell sorting staining buffer (phosphate-buffered saline, 2% fetal bovine serum or 1% bovine serum albumin, 0.1% sodium azide). All samples were incubated with anti-Fc receptor Ab (clone 2.4G2, BD Biosciences, San Jose, CA), prior to incubation with other Abs diluted in fluorescence activated cell sorting buffer supplemented with 2% anti-Fc receptor Ab. For intracellular staining, cells were collected and fixed for 50 min with 1 ml fixation buffer (IC Fixation and Permeabilization kit, eBioscience, San Diego, CA). After washing, the fixed cells were stained. The samples were filtered immediately before analysis or cell sorting to remove any clumps. The following antibodies were used: PerCP-conjugated anti-mouse B220 (Invitrogen, clone no. RA3-6B2), PE or FITC-conjugated anti-mouse CD19 (Invitrogen, clone no. MB19-1), APC-conjugated anti-mouse IgM (Invitrogen, clone no. 11/41), FITC-conjugated anti-mouse IgD (Invitrogen, clone no. 11-26c), PE-conjugated anti-mouse TACI (Invitrogen, clone no. ebio8F10-3), APC or PE-conjugated anti-mouse CD38 (Invitrogen, clone no. 90), FITC-conjugated anti-mouse GL7 (Invitrogen, clone no. GL-7), APC-conjugated anti-mouse CD138 (Invitrogen, clone no. DL-101), FITC-conjugated anti-mouse IgG1 (BD Biosciences, clone no. A85-1), PE-conjugated anti-mouse IgG2b (R&D Systems, clone no. 332723), FITC-conjugated IgG2c (LifeSpan BioSciences, cat no. LS-C349824), FITC-conjugated anti-mouse IgG3 (BD Biosciences, clone no. R40-82), eFluor 450 or PE-conjugated anti-mouse CXCR4 (Invitrogen, clone no. 2B11), and APC-conjugated anti-mouse CD86 (Invitrogen, clone no. GL1) antibodies. Naïve B cells (CD19^+^B220^+^IgM^+^IgD^+^), GC B cells (CD19^+^B220^+^GL7^+^CD38^low^), PBs (TACI^+^CD138^+^B220^int^CD19^int^), and PCs (TACI^+^CD138^+^B220^−^CD19^−^) were sorted by flow cytometry (FACS). Data collection and analyses were performed on a FACS Calibur flow cytometer using CellQuest software.

### Determination of Antibody Levels by ELISA

Antibody levels in the supernatant were determined using mouse IgM, IgG, IgG1, IgG2b, IgG2c, IgG3, IgA, and IgE (Invitrogen, Cat# 88-50470-88, 88-50400-88, 88-50410-88, 88-50430-88, 88-50670-22, 88-50440-88, 88-50450-88, and 88-50460-88, respectively) as the instructions of the manufacturers. To determine antigen-specific antibody, 96-well ELISA microtiter plates were coated with 4 μg/ml NP-ficoll or dsDNA overnight before the incubation with serially diluted sera or directly with serially diluted sera or supernatants at 4°C. Then after washing, 4 μg/ml HRP-conjugated anti-mouse IgM, IgG, IgG1, IgG2b, IgG2c, IgG3, IgA, or IgE antibodies were added to the plate and were incubated for another hour at 37°C. Finally, the color was developed by incubation with o-phenylenediamine. The OD was read at 492 nm with an ELISA reader (Bio-Rad). Standard curves were established to quantitate the amounts of the respective antibody.

### Measure of Cell Proliferation With Cell Counting Kit-8 (CCK8) Assay

Measure of cell proliferation with cell counting kit-8 (CCK8) assay was described previously (29, 33, 34). Briefly, 100 μl of cell suspension (5,000 cells/well) in a 96-well plate were cultured for an appropriate length of time (e.g., 0, 1, 2 or 3 days) in a humidified incubator (e.g., at 37°C, 5% CO_2_). Ten microliter of CCK-8 solution (Dojindo Molecular Technologies, Inc. Rockville, MD, USA) was added to each well of the plate and the plate was incubated for 1–4 h in the incubator. Measure the absorbance at 450 nm using a microplate reader.

### Co-immunoprecipitation (Co-IP)

Cells were lysised with IP Lysis Buffer (#87787, Thermo Fisher Scientific). Five hundred microliter of the supernatant from cell lysis were treated with 100 μl protein A-sepharose CL4B (Pharmacia) and incubated overnight at 4°C with constant shaking. Subsequently, the protein A-sepharose was separated by centrifugation and the resulting supernatant was incubated with 20 μl undiluted anti-Hspa13 antibody (Cat no. 12667-2-AP, Proteintech Group Inc.), and 100 μl protein A-sepharose at RT with continuous shaking for 4 h. Bound proteins as well as the sepharose matrix were then collected by centrifugation. The obtained pellet was washed 6 times with 100 μl PBS (10 mM Na-phosphate buffer, pH 7.4, 140 mM NaCl) and then collected. For gel electrophoresis, 100 μl aliquots of the supernatant samples as well as the collected pellet (cf. above) were added to 100 μl 2 × sample buffer and boiled for 2 min prior to loading. The samples were loaded in an 8% polyacrylamide gel (Bio-Rad, Hercules, CA) and subjected to SDS-PAGE electrophoresis. Silver staining was performed with Silver Staining kit (Pierce) according to the manufacturer's instructions which consisted in a standard ethanol/acetic acid fixation, ethanol washing, 30' staining with the reagents supplied, wash in ultrapure water and stop in 5% acetic acid solution. Bands were cut from the gel and digested with trypsin (Promega, Madison, WI). Enriched fraction was analyzed by LC-MS/MS that was done by H-Wayen Corp, Shanghai, China.

### Plasmid Constructs and Transfection

The recombinant plasmids expressing Hspa13-V5 and Bcap31-Flag were constructed by PCR-based amplification of Hspa13 and Bcap31 cDNA from SP 2/0 cells (ATCC® CRL-1581, Rockville, MD, USA), which was then subcloned into the pcDNA3.1 eukaryotic expression vector. The recombinant plasmids were transiently co-transfected into 293T cells with jetPEI (Polyplus Transfection) according to the manufacturer's instructions.

### Assessment of Proteinuria

Urine was manually expressed from each mouse on a weekly basis, collected into a sterile container, and assayed for the presence of protein (specifically albumin) using a colorimetric method (Albustix Reagent Strips, Bayer Corporation, Elkhart, IN).

### Statistics

Statistics were generated using *t*-test in GraphPad Prism (version 5.0, GraphPad Software Inc., USA) and values are represented as mean ± SEM. Results were considered statistically significant at *p* < 0.05.

## Results

### PBs and PCs Expressed High Levels of Hspa13

Previous studies have shown that atacicept (TACI-IgG) reduced the number of mature and activated B cells but resulted in an increase in the number of terminally differentiated PCs (16, 17). In this work, we observed that atacicept reduced the expression of PC-inhibitory genes, including Pax5 and Bcl6, and up-regulated PC-promoting genes, including Prdm1 (Blimp1) and Xbp1 (Table 1). Interestingly, Hspa13 expression was much increased in response to atacicept treatment (Table 1). These results suggest that Hspa13 mRNA levels were increased in atacicept-induced PBs and PCs.

**Table 1:** Hspa13 mRNA was increased in atacicept-induced plasmablasts (PBs) and plasma cells (PCs).

	**Gene symbol**	**Fold change**			**Regulation**
		**1st**	**2nd**	**3rd**	
GC B cell marker	Pax5	0.20	0.19	0.19	Down
	Bcl6	0.20	0.13	0.17	Down
PB/PC marker	Prdm1	14.66	20.33	15.73	Up
	Xbp1	4.88	5.40	5.89	Up
	Hspa13	6.51	9.46	6.92	Up

*Three lupus-prone MRL/lpr mice per group were injected intraperitoneally (i.p.) with 5 mg/kg atacicept (TACI-IgG) and control (IgG) at 1, 2, 3, and 4 weeks (two times per week) after mice reached 6 months of age. On day 4 after therapy, mice were euthanized and B cells were separated from the spleens by B220 microbeads. The transcripts in B cells were determined by Affymetrix Microarrays. The fold change of germinal center (GC) B cell-associated genes including Pax5 and Bcl6, PB/PC-promoting genes including Prdm1 (Blimp1) and Xbp1, and the interested gene Hspa13 (Stch) in atacicept-treated group to those in IgG group is shown. 1st, 2nd, and 3rd represent 3 independent experiments*.

**Figure 1:**
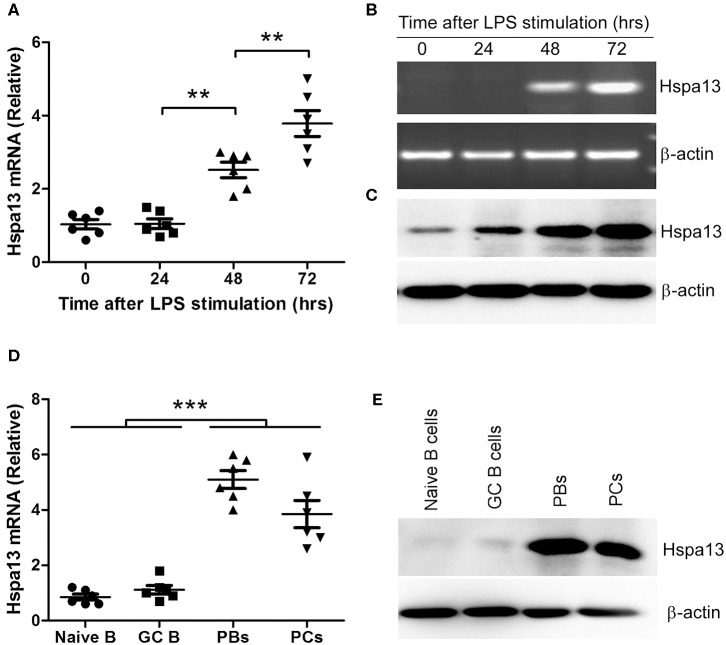
Hspa13 was highly expressed in plasmablasts (PBs) and plasma cells (PCs). **(A–C)** Hspa13 expression was up-regulated in LPS-induced PBs/PCs. The splenocytes were separated from six 7- to 9-week-old C57BL/6 mice. B cells were sorted by B220 microbeads and stimulated for 0, 24, 48, and 72 hours (hrs) *in vitro* by 10 μg/ml LPS. Cells were collected and subjected to qPCR **(A)**, RT-PCR/agarose **(B)**, and western blot **(C)** analysis. DNA band (**B**, upper panel) was verified as Hspa13 by DNA sequencing. **(D,E)** Hspa13 was highly expressed in PBs and PCs but not in naïve B cells or germinal center (GC) B cells. Lymphocytes were collected from splenocytes and lymph nodes (LNs) of six sheep red cell (SRC)-immunizated C57BL/6 mice described in Table 3. Naïve B cells (CD19^+^B220^+^IgM^+^IgD^+^), GC B cells (CD19^+^B220^+^GL7^+^CD38^low^), PBs (TACI^+^CD138^+^B220^int^CD19^int^), and PCs (TACI^+^CD138^+^B220^−^CD19^−^) were sorted by flow cytometry (FACS) and described in Supplementary Figure 1, and subjected to qPCR (D) and western blot **(E)** analysis. **(A–E)** Data represent three independent experiments with six individual mice each. **(A,D)** Data were analyzed by the one-way ANOVA plus the Bonferroni test: compare selected pairs of columns and show as mean ± s.e.m (*N* = 6 for all groups). **P < 0.01, ***P < 0.001.

To confirm that PCs expressed high levels of Hspa13 mRNA, LPS was used to induce PB and PC production *in vitro*. As expected, LPS stimulation reduced the expression of B cell-associated genes, including CD19 and Ms4a1 (CD20), and PC-inhibitory genes, including Pax5, Bcl6, and Aicda (Aid), and it up-regulated PC-promoting genes, including Prdm1 (Blimp1), Xbp1, and Hspa13 (Table 2). These results suggest that Hspa13 mRNA levels were increased in LPS-induced PBs and PCs. Furthermore, qPCR (Figure 1A), RT-PCR/agarose (Figure 1B), and western blot (Figure 1C) analysis demonstrated that LPS up-regulated Hspa13 mRNA and protein expression in a time-dependent manner.

**Table 2:** Hspa13 mRNA was increased in LPS-induced PBs/PCs.

	**Gene symbol**	**Affymetrix microarray**			**RNA-sequencing**		
		**Fold change**		**Regulation**	**None**	**LPS**	**The ratio of LPS to none**
		**1st**	**2nd**				
B-cell marker	Cd19	0.21	0.32	Down	20349.00	15874	0.78
	Ms4a1	0.43	0.52	Down	25484.96	15411	0.60
GC B cell marker	Pax5	0.09	0.29	Down	30458.14	11625	0.38
	Bcl6	0.16	0.17	Down	1865.00	403	0.22
PB/PC marker	Prdm1	34.05	27.52	Up	244.00	6055	24.82
	Xbp1	10.17	8.79	Up	2008.00	15347	7.64
	Hspa13	9.23	8.18	Up	430.00	1907	4.43

*The splenocytes were separated from three C57BL/6 mice per group. B cells were sorted by B220 microbeads and stimulated for 3 days in vitro by 10 μg/ml LPS. The transcripts in B cells and LPS-stimulated B cells were determined by Affymetrix Microarray and RNA-sequencing. The fold change of B cell-associated genes including CD19 and Ms4a1 (CD20), GC B cell-associated genes including Pax5 and Bcl6, PB/PC-promoting genes including Prdm1 and Xbp1, and the interested gene Hspa13 in LPS-stimulated group to unstimulated group is shown. 1st and 2nd represent 2 independent experiments. Total exon fragments (values) of Cd19, Ms4a1, Pax5, Bcl6, Prdm1, Xbp1, and Hspa13 in B cells (None) and LPS-stimulated B cells (LPS) are also shown*.

To further confirm that PBs and PCs expressed high levels of Hspa13 mRNA, we used single-cell RNA-sequencing to evaluate Hspa13 expression in 14 immature B cells (14.29% were positive for Hspa13 expression), 16 mature B cells (0% were positive for Hspa13 expression), 13 memory B cells (0% were positive for Hspa13 expression), 34 GC B cells (11.76% were positive for Hspa13 expression), and 10 PBs (100% were positive for Hspa13 expression) (Table 3). Only PBs expressed high levels of Hspa13, relative to immature, mature, memory, and GC B cells (Table 3). These results suggest that PBs but not naïve, memory, or GC B cells expressed high levels of Hspa13 mRNA.

**Table 3:** Hspa13 mRNA was expressed in PBs.

	**Cell numbers**	**Hspa13^**+**^ cells**	**% Hspa13^**+**^ cells**	**Hspa13 relative expression**
Immature B cells	14	2	14.29	0.11
Mature B cells	16	0	0.00	0.00
Memory B cells	13	0	0.00	0.00
GC B cells	34	4	11.76	0.07
PBs	10	10	100.00	0.59

*For sheep red cell (SRC) immunization, three 9-week-old C57BL/6 mice were i.p. injected with 1 x 10^9^ SRC and were sacrificed on day 14. Splenic B cells were sorted using B220 microbeads. The transcripts in B cells were determined by 10x Genomics and single-cell RNA-sequencing. 14 immature B cells (Cd3d^−^Cd3e^−^Cd3g^−^Cd4^−^Cd8a^−^Cd19^+^Ptprc^+^Ms4a1^+^Ighm^+^Ighd^−^Ighg1^−^Ighg2b^−^Ighg2c^−^Ighg3^−^Igha^−^Ighe^−^Bcl6^−^Aicda^−^Prdm1^−^Xbp1^−^Sdc1^−^), 16 mature B cells (Cd3d^−^Cd3e^−^Cd3g^−^Cd4^−^Cd8a^−^Cd19^+^Ptprc^+^Ms4a1^+^Ighm^+^Ighd^+^Ighg1^−^Ighg2b^−^Ighg2c^−^Ighg3^−^Igha^−^Ighe^−^Bcl6^−^Aicda^−^Prdm1^−^Xbp1^−^Sdc1^−^), 13 memory B cells (Ighg1^+^,Ighg2b^+^,Ighg2c^+^,Ighg3^+^, Igha^+^, or Ighe^+^ Cd3d^−^Cd3e^−^Cd3g^−^Cd4^−^Cd8a^−^Cd19^+^Ptprc^+^Ms4a1^+^Ighd^−^Bcl6^−^Aicda^−^ Prdm1^−^Xbp1^−^Sdc1^−^),34 GC B cells (Cd3d^−^Cd3e^−^Cd3g^−^Cd4^−^Cd8a^−^Cd19^+^Ptprc^+^Ms4a1^+^ Ighm^+^Ighd^−^Pax5^+^Bcl6^+^Aicda^+^Prdm1^−^Xbp1^−^Sdc1^−^), 10 PBs (Ighm^+^,Ighg1^+^,Ighg2b^+^,Ighg2c^+^,Ighg3^+^,Igha^+^, or Ighe^+^ Cd3d^−^Cd3e^−^Cd3g^−^Cd4^−^Cd8a^−^Cd19^+^Ptprc^+^Ms4a1^+^ Ighd^−^Bcl6^−^Aicda^−^Prdm1^+^Xbp1^+^Sdc1^+^) were chosen and Hspa13 expression was compared in these cells*.

Previous studies have shown that TACI^+^B220^+^CD138^+^ and TACI^+^B220^−^CD138^+^ were used as the markers of PBs and mature PCs, respectively (35–37). Based on these studies, naïve B cells (CD19^+^B220^+^IgM^+^IgD^+^), GC B cells (CD19^+^B220^+^GL7^+^CD38^low^), PBs (TACI^+^CD138^+^B220^int^CD19^int^), and PCs (TACI^+^CD138^+^B220^−^CD19^−^) were sorted by flow cytometry (FACS) (Supplementary Figure 1). qPCR and western blot assays suggested that Hspa13 was highly expressed in PBs and PCs but not in naïve B cells or GC B cells (Figures 1D,E).

### Reduction of PBs, PCs, and Antibodies in Hspa13 cKO Mice

**Figure 2:**
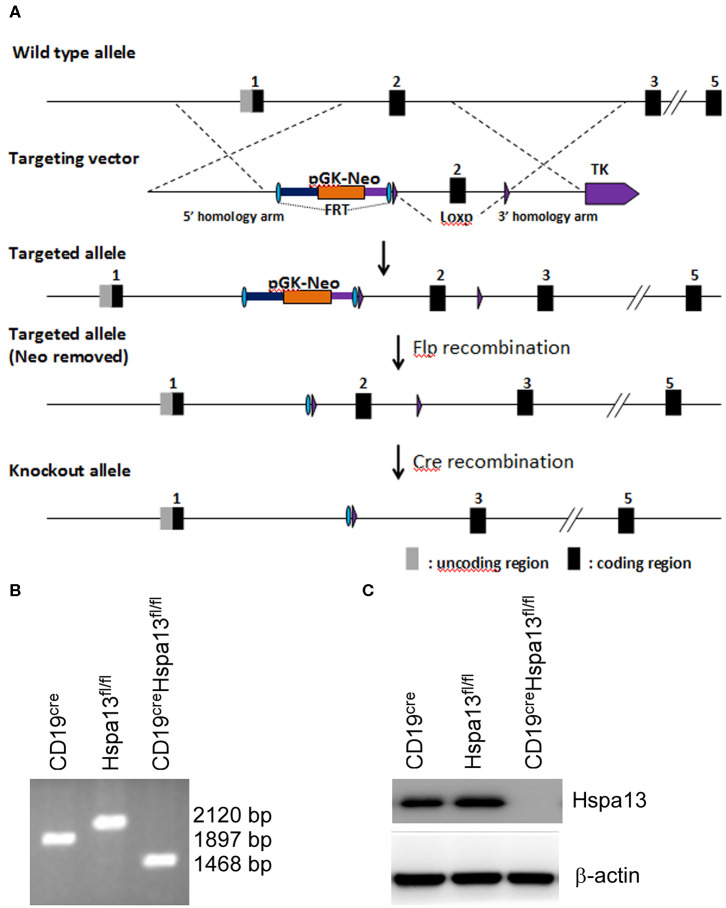
CD19^cre^Hspa13^fl/fl^ mice were developed. **(A)** A construction map of Hspa13^fl/fl^ and CD19^cre^Hspa13^fl/fl^ mice. This project used the principle of homologous recombination and adopted embryonic stem (ES) cell targeting to modify the Hspa13 locus (Chr16:75755190-75767276 bp) by flox modification. The brief process is as follows: The BAC clone containing the gene of interest was purchased from the Sanger Institute (UK). The ES cell targeting vector was constructed by the ET-clone method. The vector contains a 3.4 Kb 5′ homology arm, a 541 bp flox region, and a PGK-neo-polyA, 3.6 kb 3′ homology arm, plus MC1-TK-polyA negative selection marker. After the vector was linearized, JM8A3 ES cells were transfected electrically. A total of 96 resistant clones were obtained after screening with the G418 and Ganc drugs. A total of 13 positive clones with correct homologous recombination were identified by long fragment PCR. Positive ES cell clones were expanded and injected into blastocysts of C57BL/6J mice to obtain chimeric mice. A high proportion of chimeric mice were mated with C57BL/6J mice to obtain seven positive F1 mice. Hspa13 gene flox heterozygous mice showed no significant abnormalities. After mating the flox mouse with a heterologous CD19^cre^ mouse, the progeny of the flox homozygous, Cre-positive mouse was knocked out, resulting in a functional loss of the gene of interest in B cells. **(B)** Hspa13 was knocked out in PBs/PCs from CD19^cre^Hspa13^fl/fl^ mice. PCs (TACI^+^CD138^+^B220^−^CD19^−^) were sorted from the spleens and bone marrows (BMs) of 7- to 9-week-old heterologous CD19^cre^, Hspa13^fl/fl^, and CD19^cre^Hspa13^fl/fl^ mice by FACS. PCs were subjected to PCR (B) and western blot (C) analysis. PCR products: with cre activity: 1,468 bp; with no cre activity: 2,120 bp; wild type: 1,897 bp. **(B,C)** Data represent three independent experiments with three individual mice each.

To explore the role of Hspa13 in PBs and PCs, CD19^cre^Hspa13^fl/fl^ (B-cell specific knock-out of Hspa13, cKO) mice were developed (Figure 2A). PCs (TACI^+^CD138^+^B220^−^CD19^−^) were sorted from the spleens and bone marrows (BMs) of 7- to 9-week-old, heterologous CD19^cre^, Hspa13^fl/fl^, and CD19^cre^Hspa13^fl/fl^ mice by FACS and subjected to PCR (Figure 2B) and western blot (Figure 2C) analysis. The data demonstrated that Hspa13 was knocked out in PCs from the CD19^cre^Hspa13^fl/fl^ mouse.

**Figure 3:**
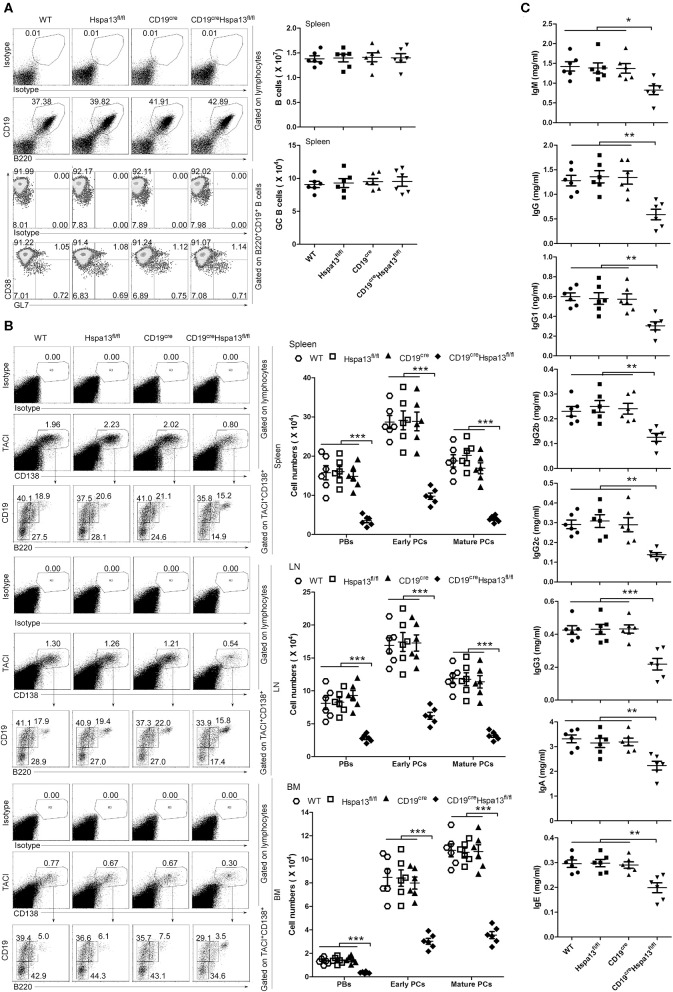
PBs, PCs, and antibodies were reduced in CD19^cre^Hspa13^fl/fl^ (Hspa13 cKO) mice. **(A)** Hspa13 cKO did not affect naïve B cells or germinal center (GC) B cells in mice. Splenic lymphocytes from 9-week-old wild type, Hspa13^fl/fl^, CD19^cre^, and CD19^cre^Hspa13^fl/fl^ mice were separated using a lymphocyte separation solution; stained with isotype control antibodies, anti-mouse CD19, B220, CD38, and GL7 antibodies; and then analyzed by FACS. The percentages (left panel) and the absolute numbers (right panel) of CD19^+^B220^+^ B cells and CD38^lo^GL7^hi^B220^+^CD19^+^ GC B cells are shown. **(B)** Hspa13 cKO reduced PBs, early PCs, and mature PCs in mice. Lymphocytes from the spleen, LNs, and BMs of 9-week-old WT, Hspa13^fl/fl^, CD19^cre^, and CD19^cre^Hspa13^fl/fl^ mice were separated using a lymphocyte separation solution; stained with isotype control antibodies, anti-mouse TACI, CD19, B220, and CD138 antibodies; and then analyzed by FACS. The percentages (left panel) and the absolute numbers (right panel) of TACI^+^CD138^+^B220^int^CD19^int^ PBs, TACI^+^CD138^+^B220^−^CD19^int^ early PCs, and TACI^+^CD138^+^B220^−^CD19^−^ mature PCs are shown. **(C)** Hspa13 cKO reduced antibodies in mice. Sera were collected from 9-week-old WT, Hspa13^fl/fl^, CD19^cre^, and CD19^cre^Hspa13^fl/fl^ mice, and the total IgM, IgG, IgG1, IgG2b, IgG2c, IgG3, IgA, and IgE antibody levels were analyzed by ELISA. **(A–C)** Data represent three independent experiments, with six mice per group per experiment. Data were analyzed by the one-way ANOVA plus the Bonferroni test: compare selected pairs of columns and show as mean ± s.e.m (*N* = 6 for all groups). *P < 0.05, **P < 0.01, ***P < 0.001.

As compared with the control group that included WT, Hspa13^fl/fl^, and CD19^cre^ mice, the Hspa13 cKO mice contained reduced levels of TACI^+^CD138^+^B220^int^CD19^int^ PBs, TACI^+^CD138^+^B220^−^CD19^int^ early PCs, and TACI^+^CD138^+^B220^−^CD19^−^ mature PCs in the spleens, lymph nodes (LNs), and BMs, but this was not the case in naïve B220^+^CD19^+^ B cells or CD38^lo^GL7^hi^B220^+^CD19^+^ GC B cells (Figures 3A,B). Accordingly, the total IgM, IgG, IgG1, IgG2b, IgG2c, IgG3, IgA, and IgE antibody levels were also reduced in Hspa13 cKO mice (Figure 3C). Collectively, these data suggest that the specific knock-out of Hspa13 in B-cells reduced the number of PBs and PCs and the levels of antibodies in mice.

### Hspa13 cKO-Mediated Reduction of the Production of Antigen-Induced PCs and Antibodies

**Figure 4:**
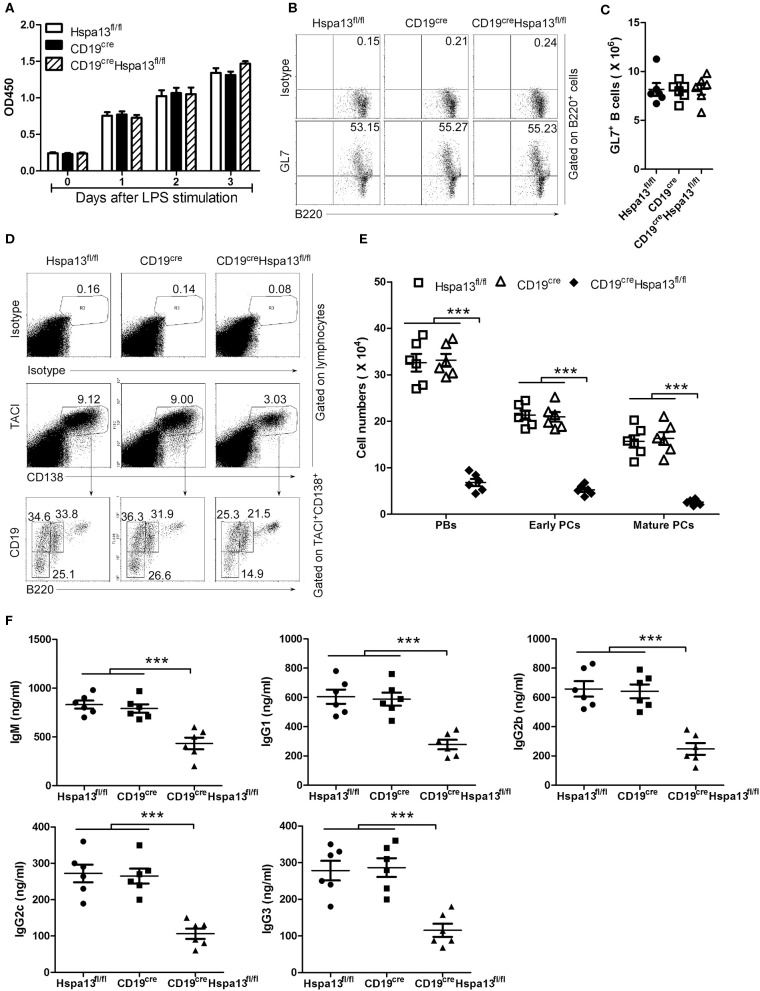
Hspa13 cKO reduced the production of LPS-induced PBs, PCs, and antibodies. Splenic B cells from 9-week-old Hspa13^fl/fl^, CD19^cre^, and CD19^cre^Hspa13^fl/fl^ mice were sorted with B220 microbeads and were then stimulated with 10 μg/ml LPS for 0, 1, 2, and 3 days. **(A)** Hspa13 cKO did not affect LPS-stimulated B-cell proliferation. On days 0, 1, 2, and 3 following LPS stimulation, a CCK8 assay was used to evaluate the cell proliferation. **(B,C)** Hspa13 cKO did not affect LPS-stimulated B-cell activation. On day 3 following LPS stimulation, cells were stained with isotype control antibodies, anti-mouse B220 and GL7 antibodies, and analyzed by FACS. The percentages **(B)** and the absolute numbers **(C)** of B220^+^GL7^+^ B cells are shown. **(D,E)** Hspa13 cKO reduced LPS-induced PBs, early PCs, and mature PCs. On day 3 following LPS stimulation, cells were stained with isotype control antibodies, anti-mouse TACI, CD19, B220, and CD138 antibodies, and were then analyzed by FACS. The percentages **(D)** and the absolute numbers **(E)** of TACI^+^CD138^+^B220^int^CD19^int^ PBs, TACI^+^CD138^+^B220^−^CD19^int^ early PCs, and TACI^+^CD138^+^B220^−^CD19^−^ mature PCs are shown. **(F)** Hspa13 cKO reduced LPS-induced antibody secretion. On day 3 following LPS stimulation, culture supernatants were collected and the total IgM, IgG1, IgG2b, IgG2c, and IgG3 antibody levels were analyzed by ELISA. **(A–F)** Data represent three independent experiments, with six mice per group per experiment. Data were analyzed by two-way **(A)** and one-way **(C,E,F)** ANOVA plus the Bonferroni test: compare selected pairs of columns and show as mean ± s.e.m (*N* = 6 for all groups). ***P < 0.001.

To explore the effect of the Hspa13 cKO on the production of antigen-induced PBs, PCs, and antibodies, a T-cell-independent antigen, LPS, was used to induce the production of PBs, PCs, and antibodies *in vitro*. The results showed that the Hspa13 cKO did not affect LPS-stimulated B-cell proliferation (Figure 4A) or the production of activated B220^+^GL7^+^ B cells (Figures 4B,C). These data suggest that the Hspa13 cKO did not affect LPS-stimulated B-cell activation. An RNA-sequencing assay showed that the Hspa13 cKO led to reduced levels of LPS-induced Prdm1 and Xbp1 mRNA (Table 4). Accordingly, the Hspa13 cKO reduced the number of LPS-induced TACI^+^CD138^+^B220^int^CD19^int^ PBs, TACI^+^CD138^+^B220^−^CD19^int^ early PCs, and TACI^+^CD138^+^B220^−^CD19^−^ mature PCs (Figures 4D,E). Thus, the Hspa13 cKO reduced the levels of LPS-induced IgM, IgG1, IgG2b, IgG2c, and IgG3 antibodies (Figure 4F). These data suggest that the Hspa13 cKO reduced the production of LPS-induced PBs, PCs, and antibodies.

**Table 4:** Hspa13 deficiency reduced Prdm1 and Xbp1 expression.

	**Gene symbol**	**Hspa13^**f/f**^**	**Hspa13 cKO**	**The ratio of Hspa13 cKO to Hspa13^**f/f**^**
	Hspa13	2,549	1,195	0.47
B-cell marker	Ptprc	30,587	31,971	1.05
	Cd19	21,222	22,334	1.05
	Ms4a1	24,949	27,911	1.12
GC B cell marker	Pax5	21,163	25,578	1.21
	Bcl6	660	590	0.89
	Aicda	2,726	2,406	0.88
PB/PC marker	Prdm1	2,294	1,288	0.56
	Xbp1	6,755	3,460	0.51
	Sdc1	28	33	1.18

*B220^+^ B cells from 7- to 9-week-old CD19^cre^Hspa13^f/f^ (Hspa13 conditional knock-out, cKO) and Hspa13^f/f^ mice were stimulated for 3 days in vitro with 10 μg/ml LPS. On day 3 following LPS stimulation, the transcripts were determined by RNA-sequencing. Total exon fragments values of the interested gene Hspa13, B cell-associated genes including Ptprc (B220), Cd19, and Ms4a1, GC B cell-associated genes including Pax5, Bcl6, and Aicda (Aid), PB/PC-promoting genes including Prdm1, Xbp1, and Sdc1 are shown*.

**Figure 5:**
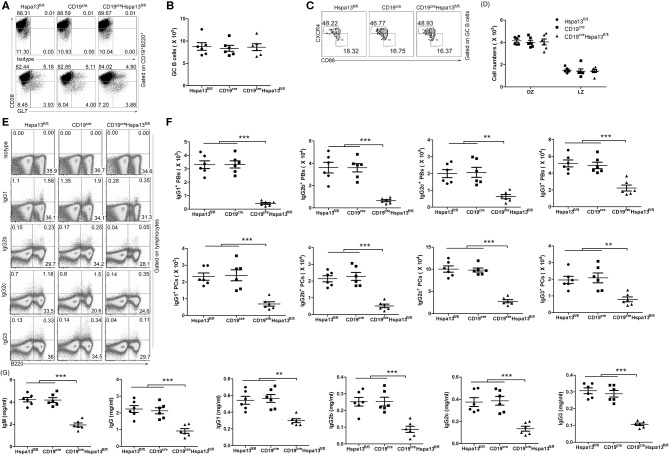
Hspa13 cKO reduced sheep red blood cell (SRC)-induced PB/PC and antibody production. Nine-week-old female Hspa13^fl/fl^, CD19^cre^, and CD19^cre^Hspa13^fl/fl^ mice were injected intraperitoneally (i.p.) with 1 × 10^9^ SRCs on days 0 and 7. **(A,B)** Hspa13 cKO did not affect SRC-induce GC B-cell production. On day 21 following SRC stimulation, splenic lymphocytes were stained with isotype control antibodies, anti-mouse CD19, B220, CD38, and GL7 antibodies, and were then analyzed by FACS. The percentages **(A)** and the absolute numbers **(B)** of CD38^lo^GL7^hi^ GC cells gated on CD19^+^B220^+^ are shown. **(C,D)** Hspa13 cKO did not affect the SRC-induced dark zone (DZ) and light zone (LZ) GC B-cell production. On day 21 following SRC stimulation, splenic lymphocytes were stained with anti-mouse CD19, B220, CD38, GL7, CXCR4, and CD86 antibodies, and were then analyzed by FACS. The percentages **(C)** and the absolute numbers **(D)** of CXCR4^hi^CD86^lo^ DZ and CXCR4^lo^CD86^hi^ LZ GC B cells gated on CD19^+^B220^+^CD38^lo^GL7^hi^ GC cells are shown. **(E,F)** Hspa13 cKO reduced SRC-induced IgG1-, IgG2b-, IgG2c-, and IgG3-expressing PBs/PCs. On day 21 following SRC stimulation, splenic lymphocytes were collected and intracellular staining was performed with isotype control antibodies, anti-mouse B220, IgG1, IgG2b, IgG2c, and IgG3 antibodies. The percentages **(E)** and the absolute numbers **(F)** of IgG1-, IgG2b-, IgG2c-, and IgG3-expressing B220^+^ PBs and B220^−^ PCs are shown. **(G)** Hspa13 cKO reduced SRC-induced antibody secretion. On day 21 following SRC stimulation, sera were collected and the total IgM, IgG, IgG1, IgG2b, IgG2c, and IgG3 antibody levels were analyzed by ELISA. **(A–G)** Data represent three independent experiments, with six mice per group per experiment. Data were analyzed by two-way **(D)** and one-way **(B,F,G)** ANOVA plus the Bonferroni test: compare selected pairs of columns and show as mean ± s.e.m (*N* = 6 for all groups). **P < 0.01, ***P < 0.001.

To assess the effect of the Hspa13 cKO on T-cell-dependent antigen-induced PBs, PCs, and antibodies, 9-week-old female Hspa13 cKO and control (Hspa13^fl/fl^ and CD19^cre^) mice were injected intraperitoneally (i.p.) with 1 × 10^9^ SRCs on days 0 and 7. We observed that the Hspa13 cKO did not affect the production of SRC-induced CD38^lo^GL7^hi^B220^+^CD19^+^ GC cells (Figures 5A,B). In addition, the Hspa13 cKO did not affect the SRC-induced dark zone (DZ) and light zone (LZ) GC B-cell production (Figures 5C,D). However, the Hspa13 cKO significantly reduced the numbers of SRC-induced IgG1-, IgG2b-, IgG2c-, and IgG3-expressing PBs and PCs (Figures 5E,F). Accordingly, the Hspa13 cKO reduced the levels of SRC-induced IgM, IgG, IgG1, IgG2b, IgG2c, and IgG3 antibodies (Figure 5G). These data suggest that the Hspa13 cKO reduced the production of SRC-induced PBs, PCs, and antibodies.

**Figure 6:**
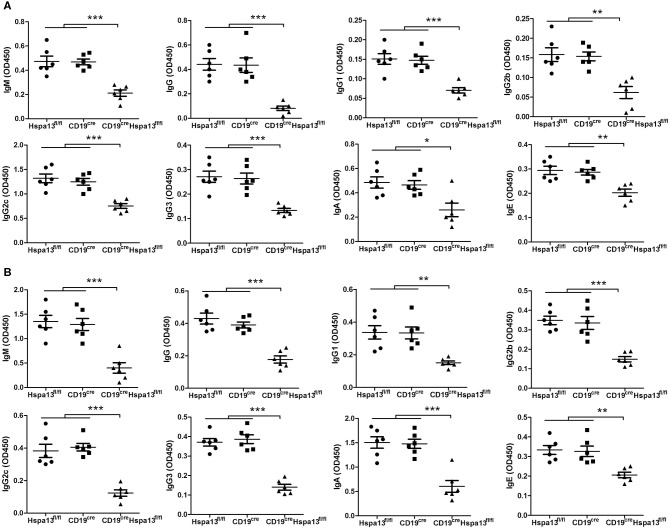
Hspa13 cKO reduced 4-hydroxy-3-nitrophenylacetyl (NP)-specific antibody production. Nine-week-old Hspa13^fl/fl^, CD19^cre^, and CD19^cre^Hspa13^fl/fl^ mice were injected i.p. with T cell-independent antigen NP-Ficoll **(A)** and T cell-dependent antigen NP-keyhole lymphocyte hemocyanin (KLH) **(B)** on days 0 and 7. On day 21 following NP-Ficoll **(A)** or NP-KLH **(B)** stimulation, sera were collected and the NP-specific IgM, IgG, IgG1, IgG2b, IgG2c, IgG3, IgA, and IgE antibody levels were analyzed by ELISA. **(A,B)** Data represent three independent experiments, with six mice per group per experiment. Data were analyzed by one-way ANOVA plus the Bonferroni test: compare selected pairs of columns and show as mean ± s.e.m (*N* = 6 for all groups). *P < 0.05, **P < 0.01, ***P < 0.001.

To assess the effect of the Hspa13 cKO on antigen-specific antibody production, 9-week-old Hspa13 cKO and control (Hspa13^fl/fl^ and CD19^cre^) mice were injected i.p. on days 0 and 7 with T cell-independent antigen (4-hydroxy-3- nitrophenyl) acetyl (NP)-Ficoll and T cell-dependent antigen NP-keyhole lymphocyte hemocyanin (KLH). We observed that the Hspa13 cKO reduced the NP-specific IgM, IgG, IgG1, IgG2b, IgG2c, IgG3, IgA, and IgE antibodies induced by NP-Ficoll (Figure 6A) and NP-KLH (Figure 6A). These results suggest that the Hspa13 cKO reduced the NP-specific antibody production.

### Hspa13 cKO Reduced Class-Switched and Somatically Hypermutated Antibody With Defective Affinity Maturation

**Figure 7:**
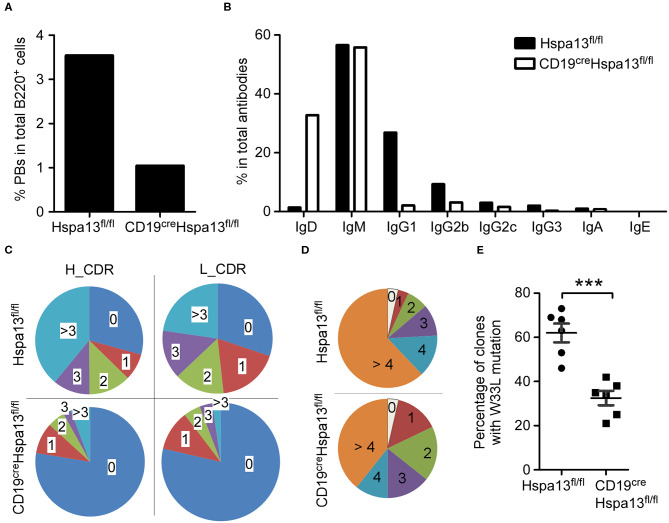
Hspa13 cKO reduced class switch recombination (CSR), somatic hypermutation (SHM), and affinity maturation of antibodies. Nine-week-old female Hspa13^fl/fl^ (control) and CD19^cre^Hspa13^fl/fl^ (Hspa13 cKO) mice (three mice per group) were injected i.p. with 1 × 10^9^ SRCs **(A–C)** or NP-KLH **(D,E)** on days 0 and 7. On day 21 following SRC stimulation, splenocytes were stained with PerCP-conjugated anti-mouse B220 antibodies and sorted by FACS. Single cells were captured using the 10 X Genomics Full Chromium platform and subjected to RNA- and VDJ-sequencing. **(A)** Hspa13 cKO reduced SRC-induced PBs. Of the single PBs, 27 (3.49%) and 11 (1.07%) (Ighm^+^, Ighg1^+^, Ighg2b^+^, Ighg2c^+^, Ighg3^+^, Igha^+^, or Ighe^+^Cd3d^−^Cd3e^−^Cd3gCd4^−^Cd8a^−^Cd19^+^Ptprc^+^Ms4a1^+^Ighd^−^Bcl6^−^Aicda^−^Prdm1^+^Xbp1^+^ Sdc1^+^) within the splenic B cell population were identified by single-cell RNA-sequencing out of 774 and 1,025 single cells corresponding to CD19^cre^Hspa13^fl/fl^ and Hspa13^fl/fl^ mice, respectively. **(B)** Hspa13 cKO reduced SRC-induced antibody CSR. Single cells expressing genes encoding IgD, IgM, IgG1, IgG2b, IgG2c, IgG3, IgA, and IgE antibodies were identified by single-cell VDJ-sequencing. The percentage of different antibody subtypes expressed by single cells out of 734 and 382 antibody-expressing single cells from CD19^cre^Hspa13^fl/fl^ and Hspa13^fl/fl^ mice, respectively, is shown. **(C)** Hspa13 cKO reduced SRC-induced antibody SHM. The single antibody gene was determined by single-cell VDJ-sequencing. SHM percentages in the CDR (complementarity-determining region) of the heavy (H) and light (L) chains are based on 382 and 734 antibody genes from Hspa13^fl/fl^ and CD19^cre^Hspa13^fl/fl^ mice, respectively. **(D)** Hspa13 cKO reduced NP-specific SHM induced by NP-KLH. The distribution of the number of mutations per unique clone (VH186.2 segment) is shown. Numbers refer to 100 individual sequences; three animals per group were analyzed. **(E)** Hspa13 cKO reduced NP-specific high-affinity clones induced by NP-KLH. On day 21 following NP-KLH stimulation, the percentage of NP high-affinity clones containing the W33L mutation in CDR1 in purified GC B cells of Hspa13^fl/fl^ and CD19^cre^Hspa13^fl/fl^ mice was determined. Each dot corresponds to a single animal (30 unique clones/mouse; Mann-Whitney test; error bars represent s.e.m; ***P < 0.001).

To assess the effect of the Hspa13 cKO on antibodies, single-cell RNA-sequencing was used to examine single PBs isolated from SRC-immunized Hspa13 cKO and control (Hspa13^fl/fl^) mice. In 774 single B220^+^ cells from Hspa13 cKO mice, we observed that 3.49% had single-cell PBs, and in 1,025 single B220^+^ cells from the control mice, we observed that 1.07% had single-cell PBs. These data suggest that the Hspa13 cKO reduced the number of SRC-induced PBs (Figure 7A). Further analysis of the IgD, IgM, IgG1, IgG2b, IgG2c, IgG3, IgA, and IgE isotypes showed that the Hspa13 cKO reduced levels of SRC-induced class-switched antibodies (e.g., IgG1, IgG2b, IgG2c, and IgG3) (Figure 7B). Furthermore, single-cell VDJ-sequencing was used to assess the somatic hypermutation (SHM) in the CDR (complementarity-determining region) of the heavy (H) and light (L) chains of 734 and 382 antibody genes from SRC-induced Hspa13 cKO and control mice, respectively. Extensive somatic mutation occurred in the CDR of the H and L chains in the controls, but it was clearly reduced in the Hspa13 cKO group (Figure 7C). These results suggest that the Hspa13 cKO reduced the SRC-induced SHM. In addition, we analyzed the NP-specific SHM in unique clones (VH186.2 segment) that had been induced by NP-KLH. Compared with the control group, Hspa13 cKO group had a lower mutational load in the V186.2 regions (Figure 7D). These results suggest that the Hspa13 cKO reduced the NP-specific SHM induced by the NP-KLH. Finally, we analyzed the NP-specific high-affinity clones from purified GC B cells that contained the W33L mutation in CDR1. The data demonstrated that the Hspa13 cKO reduced the number of NP-specific high-affinity clones induced by the NP-KLH (Figure 7E). Collectively, these data suggest that the Hspa13 cKO reduced the number of class-switched and somatically hypermutated antibodies with defective affinity maturation.

### Hspa13 Interacts With Proteins in the ER to Positively Regulate Protein Transport From the ER to the Cytosol

**Figure 8:**
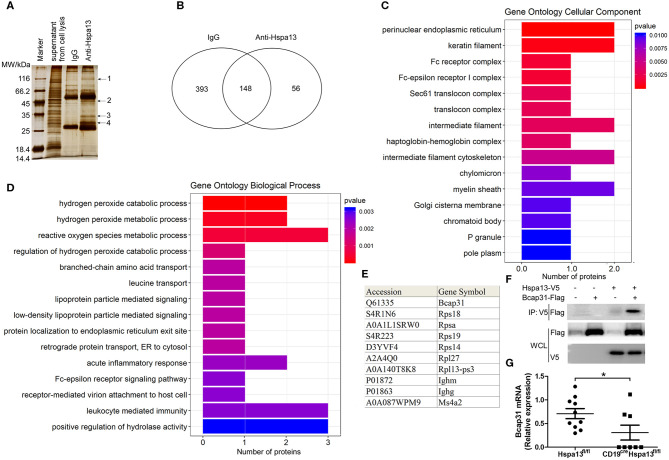
Hspa13 interacts with endoplasmic reticulum (ER) proteins involved in positive regulation of protein transport from the ER to the cytosol. Splenic B220^+^ B cells from 9-week-old C57BL/6 mice (three mice per group) were sorted using B220 microbeads and were then stimulated for 3 days with 10 μg/ml LPS. LPS-stimulated B cells were collected for anti-Hspa13 antibody co-immunoprecipitation (IP) experiments. Co-immunoprecipitated proteins were identified by mass spectrometry. **(A)** SDS-PAGE and silver staining results showing affinity captured interacting proteins from whole cell extracts. Putative interacting protein bands marked as 1, 2, 3, and 4 were excised for mass spectrometry analysis. **(B)** Anti-Hspa13 antibody specifically co-immunoprecipitated 56 proteins. We identified 393, 56, and 148 proteins that were co-immunoprecipitated by control IgG antibodies only, anti-Hspa13 antibodies only, and the two antibodies together, respectively. **(C)** Bar plot ranking of the top 10 cellular components (CC), based on enrichment score. Gene ontology (GO)-analysis was performed using a gene ontology website (http://www.geneontology.org/). **(D)** Bar plot ranking of the top 10 biologic processes (BP) based on enrichment score. GO-analysis was performed based on the gene ontology website (http://www.geneontology.org/). **(E)** A list of the 10 best hits of Hspa13 interacting partners. Detailed interacting protein data are shown in Supplementary Table 1. **(F)** Interaction of Hspa13 and Bcap31. The recombinant plasmids expressing Hspa13-V5 and Bcap31-Flag were transiently transfected into 293T cells. At 48 hrs after transfection, cells were lysed and anti-V5 antibody was used to immunoprecipitate proteins probed with anti-Flag antibody. Data are shown for one representative experiment from three independent experiments with similar results. **(G)** Hspa13 cKO reduced Bcap31 mRNA expression in PBs induced by SRC. Bcap31 mRNA expression was analyzed from 10 and 8 PBs from the single-cell RNA sequencing data of SRC-primed Hspa13^fl/fl^ and CD19^cre^Hspa13^fl/fl^ mice, respectively, described in Figure 7. Student's *t*-test (two tailed). Error bars represent s.e.m. *P < 0.05.

To explore the mechanisms underlying the production of Hspa13-regulated PBs and PCs, and antibodies, we used anti-Hspa13 antibodies to co-immunoprecipitate (IP) proteins that interact with Hspa13 in PCs induced by LPS. The results of SDS-PAGE and silver staining show affinity-captured interacting proteins from whole cell extracts (Figure 8A). Putative interacting protein bands, marked as 1, 2, 3, and 4, were excised for mass spectrometry analysis (Figure 8A). We identified 393, 56, and 148 proteins that were co-immunoprecipitated by the control IgG antibody, the anti-Hspa13 antibody, and both antibodies, respectively (Figure 8B). Gene ontology (GO)-analysis was performed based on the gene ontology website (http://www.geneontology.org/). Using the enrichment score, we determined the top 10 cellular components and biologic processes, which are listed in Figures 8C,D, respectively. GO-analysis suggests that Hspa13 interacts with proteins in the ER to positively regulate protein transport from the ER to the cytosol. The 10 best hits of the Hspa13 interacting partners are listed in Figure 8E. Detailed interacting protein data are shown in Supplementary Table 1. The expression of the most interesting target Bcap31 has been confirmed at the protein level by western blotting and co-IP experiments with a tagged, transfected target gene (Figure 8F). Finally, the Bcap31 mRNA expression was analyzed from the single-cell RNA sequencing data of the SRC-primed Hspa13^fl/fl^ mice (10 PBs) and CD19^cre^Hspa13^fl/fl^ mice (8 PBs) (Figure 7). The results suggest that the Hspa13 cKO reduced the Bcap31 mRNA expression (Figure 8G). Collectively, our data suggest that Hspa13 interacts with proteins (e.g., Bcap31) in the ER to positively regulate protein transport from the ER to the cytosol.

### Increased Hspa13 Expression in B220^+^ Cells From Patients With MM or SLE

To explore the Hspa13 expression in PC-related diseases, two such diseases (MM and SLE) were studied in this work. CD19^+^ cells from peripheral blood monocytes of healthy donors and patients with MM or SLE were sorted using CD19 microbeads, and an RNA-sequencing assay was used to determine the transcript sequences. The results demonstrated that PC-promoting genes including Xbp1 and Sdc1 (Cd138) were increased in patients with MM (Table 5) or SLE (Table 6). These data suggest that there were more PCs in MM patients. Accordingly, we found that Hspa13 mRNA was also increased in B220^+^ cells from patients with MM (Table 5) or SLE (Table 6).

**Table 5:** Hspa13 mRNA was increased in B220^+^ cells from patients with multiple myeloma (MM).

		**Total exon fragments**			**FPKM**		
	**Gene symbol**	**Health**	**MM**	**The ratio of MM to health**	**Health**	**MM**	**The ratio of MM to health**
B-cell marker	Ptprc	28,233	23,376	0.83	212.69	170.13	0.80
	Cd19	329	47	0.14	7.78	1.07	0.14
	Ms4a1	4,026	542	0.13	52.96	6.89	0.13
GC B cell marker	Pax5	914	149	0.16	6.33	1	0.16
	Bcl6	1,934	2,891	1.49	20.87	30.14	1.44
	Aicda	3	1	0.33	0.06	0.02	0.33
PB/PC marker	Prdm1	1,044	1,197	1.15	10.5	11.64	1.11
	Xbp1	2,303	4,256	1.85	48.74	87.02	1.79
	Sdc1	9	125	13.89	0.16	2.15	13.44
	Hspa13	470	726	1.54	7.18	10.72	1.49

*CD19^+^ cells from peripheral blood monocytes of three healthy donors and three patients with MM were sorted by CD19 microbeads. The transcripts were determined by RNA-sequencing. Total exon fragments and FPKM (FPKM = total exon fragments/mapped reads (millions)/exon length (KB), Fragments per Kilo bases per Million reads) values of B cell-associated genes including Ptprc, Cd19, and Ms4a1, GC B cell-associated genes including Pax5, Bcl6, and Aicda, PB/PC-promoting genes including Prdm1, Xbp1, and Sdc1 and the interested gene Hspa13 are shown*.

**Table 6:** Hspa13 mRNA was increased in B220^+^ cells from patients with systemic lupus erythematosus (SLE).

		**Health**		**SLE**		**The ratio of SLE to health**
	**Gene symbol**	**1st**	**2nd**	**1st**	**2nd**	
B-cell marker	Ptprc	26,506	26,733	29,061	18,529	0.89
	Cd19	475	421	810	170	1.09
	Ms4a1	5,084	3,502	5,787	1,312	0.83
GC B cell marker	Pax5	1,434	1,547	2,865	349	1.08
	Bcl6	4,548	2,211	5,788	8,721	2.15
	Aicda	0	0	3	0	
PB/PC marker	Prdm1	2,442	2,972	4,048	2,232	1.16
	Xbp1	4,691	4,374	3,421	4,311	0.85
	Sdc1	2	7	22	11	3.67
	Hspa13	533	661	891	1,434	1.95

*CD19^+^ cells from peripheral blood monocytes of three healthy donors and three patients with SLE were sorted by CD19 microbeads. The transcripts were determined by RNA-sequencing. Total exon fragments values of B cell-associated genes including Ptprc, Cd19, and Ms4a1, GC B cell-associated genes including Pax5, Bcl6, and Aicda, PB/PC-promoting genes including Prdm1, Xbp1, and Sdc1 and the interested gene Hspa13 are shown. 1st and 2nd represent 2 independent experiments*.

**Figure 9:**
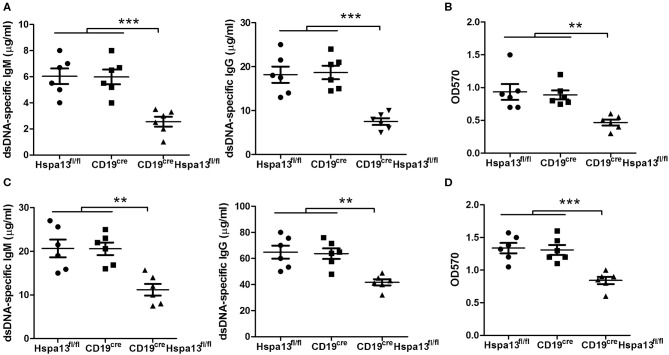
Hspa13 cKO reduced autoantibodies and proteinuria in pristane-induced lupus and lupus-prone MRL/lpr mouse model. **(A)** Hspa13 cKO reduced autoantibodies in pristane-induced lupus mice. To induce lupus, 9-week-old female Hspa13^fl/fl^, CD19^cre^, and CD19^cre^Hspa13^fl/fl^ mice on a B6 background were injected i.p. with 0.5 ml of pristane. On day 21 following pristane stimulation, sera were collected and dsDNA-specific IgM and IgG antibody levels were analyzed by ELISA. **(B)** Hspa13 cKO reduced proteinuria in the pristane-induced lupus mice. On day 21 following pristane stimulation, urine was collected and proteinuria was measured. **(C)** Hspa13 cKO reduced autoantibodies in a lupus-prone MRL/lpr mouse model. Sera were collected from Hspa13^fl/fl^, CD19^cre^, and CD19^cre^Hspa13^fl/fl^ mice in lupus-prone MRL/lpr mice background at 6 months of age and dsDNA-specific IgM and IgG antibody levels were analyzed by ELISA. **(D)** Hspa13 cKO reduced proteinuria in the lupus-prone MRL/lpr mouse model. Urine was collected from Hspa13^fl/fl^, CD19^cre^, and CD19^cre^Hspa13^fl/fl^ mice in a lupus-prone MRL/lpr mice background at 6 months of age and proteinuria was measured. **(A–D)** Data represent three independent experiments, with six mice per group per experiment. Data were analyzed by the one-way ANOVA plus the Bonferroni test: compare selected pairs of columns and show as mean ± s.e.m (*N* = 6 for all groups). **P < 0.01, ***P < 0.01.

To explore the role of Hspa13 in SLE, two lupus mouse models were used. The hydrocarbon oil 2, 6, 10, 14-tetramethylpentadecane (TMPD; also known as pristane)-induced experimental lupus mice displayed some important immunologic and clinical features that are similar to those in human SLE (38–40). We found that Hspa13 cKO reduced the number of autoantibodies (Figure 9A) and level of proteinuria (Figure 9B) in the mouse model with pristane-induced lupus. MRL/lpr mice are considered a good spontaneous model of human SLE diseases (41, 42). We found that Hspa13 cKO reduced the number of autoantibodies (Figure 9C) and the level of proteinuria (Figure 9D) in the lupus-prone MRL/lpr mouse model. Collectively, our data suggest that Hspa13 was increased in PC-associated diseases (e.g., MM and SLE), whereas the B-cell-specific KO of Hspa13 reduced the production of autoantibodies and proteinuria in the lupus mouse model.

## Discussion

PCs play an important role in both MM and SLE. However, there is still not an effective way to control PCs. In this work, we showed that Hspa13 was increased in B220^+^ B cells from patients with MM or SLE. PBs and PCs (but not naïve B cells or GC B cells) expressed high levels of Hspa13. The B-cell-specific KO of Hspa13 reduced the production of PBs and PCs, and the secretion of antibodies. These results suggest that patients with PC-associated diseases (e.g., MM and SLE) may benefit from treatments based on Hspa13.

Published reports regarding the role of HSPs in PCs are limited. One previously published paper showed that Hsp90 inhibitors induced the UPR to reduce abnormal immunoglobulin production and finally resulted in myeloma cell death (26). In this work, we found that the B-cell specific knock-out of Hspa13 reduced the numbers of PBs and PCs and the levels of antibodies in mice (Figure 3). In addition, the Hspa13 cKO reduced the PB, PC, and antibody production induced by LPS (Figure 4), SRCs (Figure 5), and NP-Ficoll and NP-KLH (Figure 6). Collectively, these data demonstrated that the Hspa13 cKO reduced the production of PBs, PCs, and antibodies, suggesting that Hspa13 may be an effective target for PCs.

We found that 100% of PBs expressed Hspa13, whereas only a few immature, mature, memory, and GC B cells expressed low levels of Hspa13 (Table 3). In addition, Hspa13 mRNA and protein were highly expressed in PBs and PCs but not in naïve B cells or GC B cells (Figures 1D,E). These results suggest that PBs and PCs expressed high levels of Hspa13 mRNA. Since the discovery of Hspa13 (Stch) in 1994 (43), only PBs and PCs have been shown to express Hspa13. This suggests that targeting Hspa13 in PBs and PCs may not result in serious side effects.

Our present study and previous studies demonstrated that atacicept (TACI-IgG) (16, 17) and LPS (29, 30, 40) resulted in an increase of terminally differentiated PCs. In this work, we found that the expression of PC-promoting genes, including Prdm1 (Blimp1) and Xbp1, and of Hspa13, was up-regulated by atacicept (Table 1) and LPS (Table 2), whereas the Hspa13 cKO reduced the levels of LPS-induced Prdm1 and Xbp1 mRNA (Table 4). Because PBs and PCs express high levels of Hspa13, Hspa13 expression may be positively associated with the PC-promoting genes Prdm1 and Xbp1. In fact, when the numbers of PBs and PCs are reduced, the Hspa13 is also reduced, and in turn, when the Hspa13 is reduced, the number of PCs is reduced.

High-affinity antibody production is a critical step in long-term immune responses (44). The mediation of SHM and CSR by activation-induced cytidine deaminase (Aicda, AID) is an important step in the generation of high-affinity responses (45). Following B-cell activation, CSR of IgM into IgG, IgE, or IgA occurs rapidly (46, 47). All of the SHM, CSR, and affinity maturation occurs in the germinal center (48). We found that Hspa13 was not expressed in GC B cells (Tables 1–3, and Figure 1) and that the Hspa13 cKO did not affect B-cell activation or GC B-cell production (Figures 3–5). However, the Hspa13 cKO reduced the class-switched (Figure 7B) and somatically hypermutated (Figures 7C,D) antibodies with defective affinity maturation (Figure 7E). This may have been because the Hspa13 cKO reduced the production of PBs and PCs (Figures 3–5).

A co-IP assay was used to identify 56 proteins that interact with Hspa13 (Figure 8 and Supplementary Table 1). GO-analysis and co-IP experiments (Figure 8) suggest that Hspa13 interacts with proteins (e.g., Bcap31) in the ER to positively regulate protein transport from the ER to the cytosol. Our results are consistent with previous studies that suggested that Hspa13 (Stch) belongs to the HSP70 family with ATPase that aids in the production of cytosolic and secretory proteins (43). Importantly, our experiments also demonstrated that Hspa13 regulates PB, PC, and antibody production (Figures 3–6). These results suggest that Hspa13 regulates the production of PBs, PCs, and antibodies by regulating the protein (e.g., antibody) transport from the ER to the cytosol.

To explore the role of Hspa13 in PC-related diseases, we used an RNA-sequencing assay to evaluate Hspa13 expression. The results demonstrated that the expression of PC-promoting genes, including Xbp1 and Sdc1 (Cd138), was increased in patients with MM (Table 5) or SLE (Table 6). Accordingly, we also found that the Hspa13 mRNA was increased in B220^+^ cells from patients with MM (Table 5) or SLE (Table 6). Critically, the Hspa13 cKO reduced the number of autoantibodies and the level of proteinuria in the pristane-induced lupus and lupus-prone MRL/lpr mouse models (Figure 9). Autoantibodies have been shown to be related to pathologic features of SLE such as lymphopenia and proteinuria (49). This suggests that targeting of the Hspa13 may be a productive strategy for treating patients with PC-related diseases (e.g., SLE).

In conclusion, PBs and PCs expressed high levels of Hspa13, which was increased in MM and SLE, whereas the B-cell-specific KO of Hspa13 reduced the production of PBs and PCs and the secretion of antibodies. Thus, the targeting of Hspa13 may be a productive strategy for treating patients with PC-related diseases (e.g., SLE).

## Data Availability Statement

The generated and/or analyzed datasets of the current study are available in the ArrayExpress repository, http://www.ebi.ac.uk/arrayexpress/experiments/E-MTAB-7106, 7109, 8280, 7112, 7146, and 7145.

## Ethics Statement

The animal study was reviewed and approved by the Animal Ethics Committee of the Beijing Institute of Basic Medical Sciences.

## Author Contributions

YH, RX, BZ, YF, CH, and CX performed the experiments. HX, GC, XW, NM, and GH contributed essential reagents and materials for the experiments. RW conceived of and designed the studies. RX and RW contributed to data analysis and manuscript preparation. All authors have read and approved the final manuscript.

## Conflict of Interest

XW was employed by the company Staidson (Beijing) Biopharmaceuticals. The remaining authors declare that the research was conducted in the absence of any commercial or financial relationships that could be construed as a potential conflict of interest.

## Supplementary Material

Supplementary Table 1 The interacting proteins with Hspa13.
