# Supplementary material for: Hspa13 Promotes Plasma Cell Production and Antibody Secretion
Source: Front Immunol. 2020 May 29;11:913. doi: 10.3389/fimmu.2020.00913 (PMC7272575; doi:10.3389/fimmu.2020.00913)
Supplement: Supplementary file 1 [file Image_1.pdf]

Supplementary Figure 1

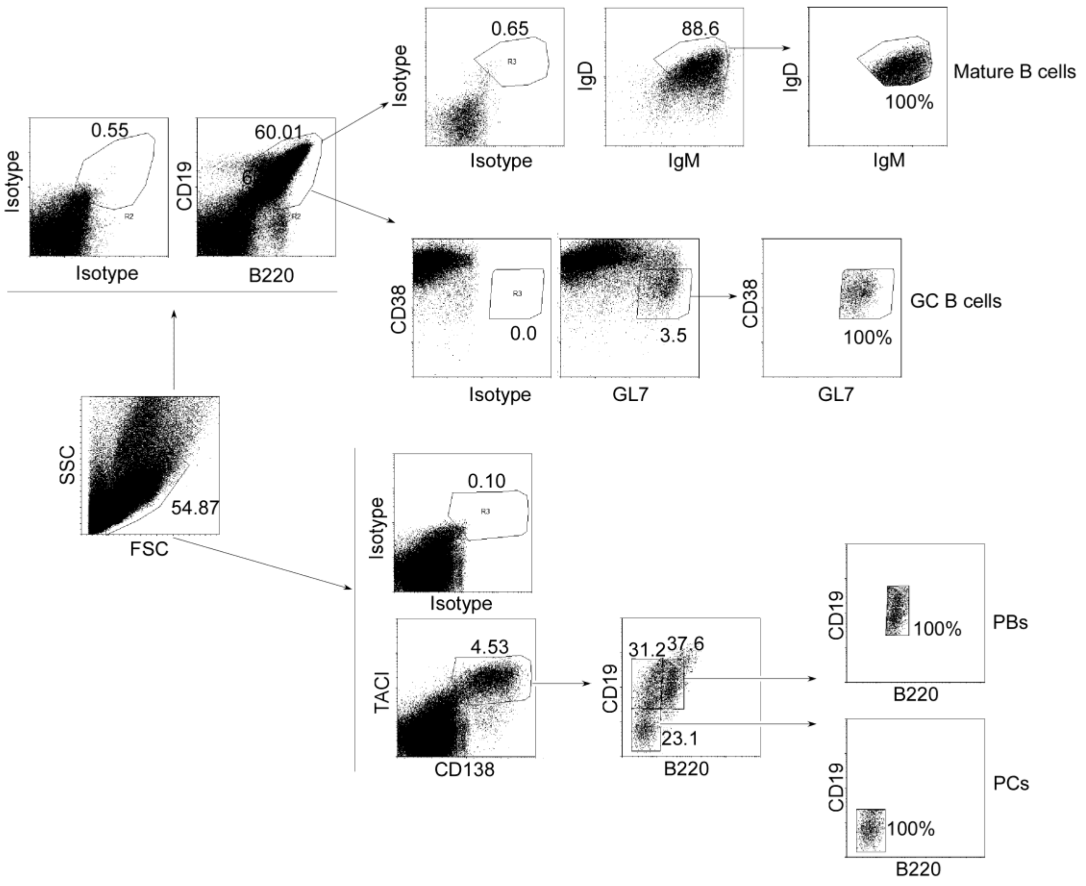

Supplementary Figure 1. The gating strategy for analysis and sorting of splenic naïve B cells, germinal center (GC) B cells, plasmablasts (PBs), and plasma cells (PCs) from sheep red cell (SRC)-immunizated C57BL/6 mice described in Table III and Figure 1D, E. Naïve B cells: $CD19^{+}B220^{+}IgM^{+}IgD^{+}$, GC B cells: $CD19^{+}B220^{+}GL7^{+}CD38^{low}$, PBs: $TACI^{+}CD138^{+}B220^{int}CD19^{int}$, PCs: $TACI^{+}CD138^{+}B220^{-}CD19^{-}$.
